# Socioeconomic status and depressive symptoms among older adults in China: The mediating role of cognitive function, lifestyle and social participation

**DOI:** 10.1371/journal.pone.0341370

**Published:** 2026-02-10

**Authors:** Ying Qin, Xiyu Liu, Nan Wang, Huimin Liu, Qianyu Zhou, Jiajun Chen, Mengting Liu, Changqing Sun, Hui Wang, Dandan Liu

**Affiliations:** 1 School of Nursing and Health, Zhengzhou University, Zhengzhou, China; 2 Institute for the Prevention and Control of Sexually Transmitted Diseases and AIDS, Henan Provincial Center for Disease Control and Prevention, Zhengzhou, China; 3 School of Public Health, Zhengzhou University, Zhengzhou, China; 4 Hospital Infection Management Department, Yuncheng Central Hospital, Yuncheng, China; 5 Medical Department, First People’s Hospital of Zhengzhou, Zhengzhou, China; University of Sao Paulo, BRAZIL

## Abstract

**Introduction:**

Socioeconomic status (SES) is a key risk factor for depression in older adults, while cognitive function, lifestyle and social participation also have an impact on depression. This study aimed to investigate the mediating role of cognitive function, lifestyle and social participation in the association between SES and depressive symptoms among older adults in China.

**Methods:**

Data were derived from the Chinese Longitudinal Healthy Longevity Survey (CLHLS) (2017−2018). A total of 7595 community-dwelling adults aged ≥65 years were included. Depressive symptoms were assessed using the 10-item Center for Epidemiologic Studies Depression Scale (CES-D-10). SES was measured as a composite index incorporating education level, occupation, and self-rated economic status. Cognitive function was evaluated via the Mini Mental State Examination (MMSE). Lifestyle and social participation scores were constructed based on relevant questionnaire items. Mediation analysis was performed to explore the indirect effects of cognitive function, lifestyle, and social participation on the association between SES and depressive symptoms.

**Results:**

The prevalence rate of depressive symptoms (CES-D-10 score ≥10) was 41.1%. After adjusting for sociodemographic and health-related covariates, SES was negatively associated with depressive symptoms (β = −0.887, *P* < 0.001). SES had a significant mediating effect on depression in older adults respectively, through cognitive function (relative mediating effect = 8.0%, β = −0.071, 95%CI: −0.095 ~ −0.048), lifestyle (19.9%, β = −0.177, 95%CI: −0.213 ~ −0.140) and social participation (7.6%, β = −0.068, 95%CI: −0.095 ~ −0.042). Additionally, sequential mediating effects were observed for “cognitive function → lifestyle” (1.0%, β = −0.009, 95%CI: −0.012 ~ −0.006), “cognitive function → social participation” (1.0%, β = −0.009, 95%CI: −0.014 ~ −0.006), “lifestyle → social participation” (1.4%, β = −0.012, 95%CI: −0.018 ~ −0.007), and “cognitive function → lifestyle → social participation” (0.1%, β = −0.001, 95%CI: −0.001 ~ −0.001).

**Conclusion:**

SES influences depressive symptoms in Chinese older adults through both direct and indirect pathways. The findings highlight the need for multifaceted interventions targeting cognitive function enhancement, healthy lifestyle promotion, and social participation facilitation, particularly among socioeconomically disadvantaged older populations, to mitigate depressive symptoms and promote healthy aging.

## Introduction

Population aging will be the basic national condition of China for a long time in the future. It is also an irreversible economic and social normal phenomenon, which is changing the population basis of national development. According to the data of the seventh national population census, the number of elderly people aged 65 and above in China has reached 191 million, accounting for 13.5% of the total population. This represents an increase of 4.63 percent compared with the sixth census [1]. By 2050, China’s elderly population is expected to exceed 300 million, accounting for about 30 percent of the total population [2]. With the acceleration of population aging, the problem of “aging before health care” has become increasingly prominent. Depression is one of the common mental health problems in older adults. The World Health Organization reported that depression affects about 7% of older people worldwide [3]. In China, the pooled overall prevalence of depressive symptoms among older adults is 20.0% [4]. Depression, as a clinical disorder, is characterized by persistent sadness, self-blame, loss of interest in daily activities, and other negative emotions, and it has well-documented adverse effects on both physical and mental health outcomes [5,6]. However, in epidemiological studies, it is often not feasible to rely on clinical diagnoses; instead, depressive symptoms—observable manifestations of low mood, anhedonia, and related feelings—are assessed through validated scales. In older adults, these depressive symptoms can accelerate the progression of existing diseases, elevate the risk of suicide, and impose a considerable burden on families and society [7].

Depression in older adults is often caused by many factors, including multifactorial correlates of biological, psychosocial, and environmental factors. Socioeconomic status (SES) is an important indicator of social structure. It reflects the position of an individual or group within a class-based society. SES is also a comprehensive measure that incorporates education, income, occupation, wealth, and residential area. Due to the differences in economic development and medical resource allocation between urban and rural areas and between eastern and western regions in China, the elderly population has obvious differences in physical health, mental health, average life expectancy and death risk. It is closely related to depressive symptoms in older adults, and it is also one of the important factors affecting depressive symptoms in older adults. Many previous studies have shown that there is an inverse association between SES and depressive symptoms in older adults [8,9]. Compared with people with high SES, people with low SES have lower control over their work and life, face adverse events or challenges more frequently, and are more likely to experience negative events, which may lead to potential psychosocial and behavioral harm. Long-term exposure to such social stressors may contribute to the development of depressive symptoms [10,11].

Previous studies have shown that there is a positive correlation between SES and cognitive function in older adults, and older adults with low socioeconomic status have lower cognitive function [12,13]. According to the active cognitive reserve theory, people with higher education have better cognitive function, and this phenomenon will continue with age, thereby slowing the progression of dementia [14]. At the same time, there is a complex relationship between cognitive function and depression in older adults. Elderly patients with depression are accompanied by cognitive impairment, and patients with cognitive dysfunction are often accompanied by depression [15]. Therefore, it can be speculated that cognitive function is one of the intermediate links between SES and depression in older adults. Similarly, lifestyle and social participation may also play a role in the association between SES and depression in older adults. Previous studies have pointed out that people with higher SES tend to have a favorable lifestyle [16], while unhealthy lifestyles such as smoking and excessive drinking are risk factors for depression in older adults [17]. Zhang found that social participation mitigated the adverse health effects of disadvantaged SES on older adults [18]. Therefore, the following hypotheses were proposed in this study: (1) SES affects depression in older adults by influencing their cognitive function; (2) SES affects depression in older adults by influencing their lifestyle; (3) SES affects depression in older adults by influencing their social participation.

Few previous studies have explored the effect of SES on depression from both physiological and behavioral aspects in the elderly. Moreover, previous studies mostly explored the role of single variable between SES and depression. Therefore, this study aimed to examine the association between SES and depressive symptoms in older adults. It further explored the mediating role of cognitive function, lifestyle and social participation in this association by using population-based data of elderly Chinese people. We believe our study can provide a scientific basis for reducing the incidence of depressive symptoms in older adults, improving the mental health of older adults, and promoting healthy aging.

## Materials and methods

### Samples

The data for this study were obtained from the Chinese Longitudinal Healthy Longevity Survey (CLHLS) [19]. CLHLS collected longitudinal data coordinated by the Center for Healthy Aging and Development Studies of National School of Development at Peking University. The CLHLS aims to investigate factors influencing healthy aging in China, with baseline data collected in 1998 and follow-up surveys conducted every 2–3 years. The 2017–2018 wave included participants from 23 provinces, covering approximately half of the counties and urban districts in each province, with a stratified random sampling design ensuring national representativeness.

**Fig 1:**
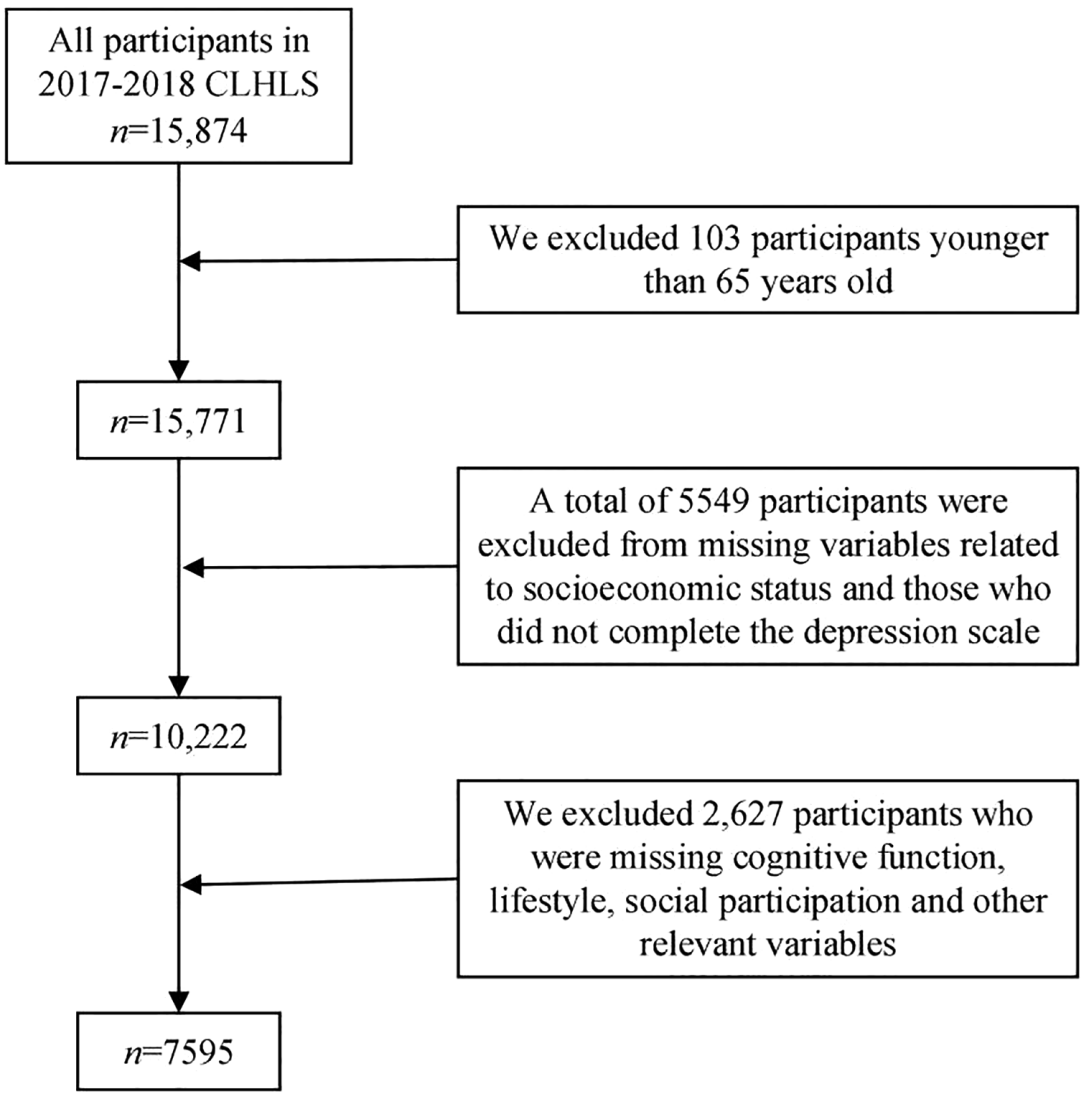
Research object screening flow chart.

The questionnaire data collected provides information on family structure, living arrangements and proximity to children, activities of daily living (ADL), the capacity of physical performance, self-rated health, self-evaluation of life satisfaction, cognitive functioning, chronic disease prevalence, care needs and costs, social activities, diet, smoking and drinking behaviors, psychological characteristics, economic resources, and caregiving and family support among elderly respondents and their relatives [20]. All the survey data are publicly available. The original 2017–2018 CLHLS dataset included 15,874 participants. For the present study, we applied the following inclusion criteria: (1) aged ≥65 years; (2) complete data on key variables (SES, depressive symptoms, cognitive function, lifestyle, social participation); and (3) no logical inconsistencies in responses. Exclusion criteria included: (1) duplicate records; (2) missing data on ≥1 key variable; and (3) outliers (defined as values ±3 standard deviations from the mean for continuous variables). A total of 7,595 participants were included in the final analysis. The selection process of research objects is shown in Fig 1. The samples were well representative.

### Variables and instruments

#### Dependent variable: Depressive symptoms.

Depressive symptoms were assessed using the 10-item Center for Epidemiologic Studies Depression Scale (CES-D-10). The CES-D-10 measures the frequency of depressive symptoms (e.g., “I felt sad,” “I had trouble falling asleep”) over the past week, using a 4-point Likert scale. Two positive items (“I was happy” and “I felt hopeful about the future”) were reverse-coded prior to summation. Total scores range from 0 to 30, with higher scores indicating more severe depressive symptoms. A cutoff score of ≥10 was used to define clinically relevant depressive symptoms, consistent with previous studies in Chinese older populations [21]. Previous studies have confirmed the reliability and validity of CESD-10 in this sample [22].

#### Independent variable: Socioeconomic status.

Socioeconomic status (SES) is one of the most widely studied constructs in the social sciences [23]. We measured SES as a composite index integrating three key indicators: education level, occupation, and self-rated economic status. Each indicator was coded as follows:

Education level: 1 = illiterate (no schooling), 2 = primary/junior high school (1–9 years), 3 = high school or above (≥10 years).

Occupation (prior to age 60):1 = general practitioners (freelancers, farmers, domestic workers, unemployed), 2 = intermediate practitioners (general staff, service workers, laborers), 3 = senior practitioners (professional/technical personnel, doctors, teachers, administrators, military).

Self-rated economic status: 1= poor, 2= fair, 3=good.

SES composite scores ranged from −1.46 to 2.59, with higher scores indicating a higher socioeconomic status of the participants [24].

#### Mediating variables: Cognitive function.

Assessed using the Mini-Mental State Examination (MMSE), a widely used tool for evaluating global cognitive function. The scale included 24 items in five domains: general ability, reaction ability, attention and calculation ability, recall ability and language ability, understanding and self-coordination ability. The total score ranged from 0 to 30, with higher scores indicating better cognitive function. The validity and reliability of the MMSE scale have been verified in several previous studies [25–27].

#### Lifestyle.

The lifestyle score was constructed based on five health-related behaviors: diet, smoking, alcohol consumption, exercise, and sleep.

Diet: Two items (“Do you often eat fresh vegetables?” and “Do you often eat fresh fruit?”) were coded as 1 = “every day/almost every day” or “often,” 0 = “rarely/never” or “sometimes.”

Smoking: 1 = non-smoker, 0 = current smoker.

Alcohol consumption: 1 = non-drinker, 0 = current drinker.

Exercise: 1 = regular exercise (≥1 time/week), 0 = infrequent exercise (<1 time/week).

Sleep duration: 1 = 7–9 hours/night, 0 = <7 hours or >9 hours/night.

The total score of six items was used to reflect the lifestyle of the participants, with higher scores indicating a healthier lifestyle for older adults [28].

#### Social participation.

Previous studies have shown that the social participation of the elderly in China is more reflected in leisure and entertainment, daily exercise and family care [29]. Therefore, we divided the social participation into two dimensions based on eight items: self-recreation and group interaction.

Self-recreation: Doing housework, gardening/pet care, reading newspapers/books, raising poultry/livestock, watching TV/listening to the radio.

Group interaction: Outdoor activities, playing cards/mahjong, participating in organized social events. Responses were coded on a 5-point scale (1 = almost every day, 2 = at least once a week, 3 = at least once a month, 4 = sometimes, 5 = never), with reverse coding applied to ensure higher scores indicate greater participation [30].

#### Covariates.

Covariates were selected based on previous literature on late-life depression and included:

Sociodemographic variables: Age (65–74, 75–84, 85–94, ≥ 95 years), gender (male/female), residence (urban/rural), marital status (with spouse/without spouse).

Health-related variables: Activities of daily living (ADL; impaired/normal, assessed using the Barthel Index), self-rated health (good/fair/poor), hearing status (normal/difficult), and history of serious illness in the past two years (yes/no).

### Statistical analysis

Stata 16.0 software was used to clean the 2017–2018 CLHLS data, and SPSS 21.0 was used for statistical analysis. Pearson’s *χ*^2^ test was used to compare categorical variables between participants with and without depressive symptoms. The SPSS macro program (PROCESS) developed by Hayes [31] was used for mediating effect analysis to explore the mediating effect. The biasing corrected percentile Bootstrap method (repeated sampling 5000 times) and 95% Confidence Interval (95%CI) were used to infer the significance of the mediating effect. If the 95%CI did not include 0, the mediating effect was significant. We conducted mediation analyses separately using “cognitive function”, “lifestyle”, and “social participation” as mediating variables. In addition, “cognitive function-lifestyle”, “cognitive function-social participation”, and “cognitive function-life-style -social participation” were used as the chain mediators for the mediation analysis. To verify the robustness of our findings, we conducted sensitivity analyses. Considering the significant urban-rural dual structure and the differences between men and women, we performed stratified analyses to test whether the mediation models remained consistent. Statistical significance was set at P < 0.05.

### Ethics approval and consent to participate

The data were obtained from a publicly accessible database of the Chinese Longitudinal Healthy Longevity Survey with a signed data use agreement. The CLHLS was approved by research ethics committees of Peking University and written informed consents from all participants or their representatives were collected.

## Results

### Characteristics of the study sample

Participants’ characteristics are shown in Table 1. Among the 7595 participants, the mean age was 82.2 years old (16.2% aged 100 and above), and males accounted for about 46.2%. 58.4% of the participants lived in cities and 50.0% had spouses. 15.0% of the participants had impaired activities of daily living. The proportion of participants with good, fair and poor self-rated health status was 48.9%, 38.6% and 12.5%, respectively. Participants with hearing difficulties accounted for 68.9%. In the past two years, 72.5% of them suffered from severe illness.

**Table 1:** The characteristics of depressive symptoms in Chinese adults aged 65 years and older (*n* = 7595).

Variables	*n*	Percent (%)	Have depression symptoms		No depression symptoms		*χ* ^2^	*P*
			*n*	Percent (%)	*n*	Percent (%)		
**Age group, years**								
65~	2215	29.2	786	35.5	1429		45.533	<0.001
75~	2333	30.7	973	41.7	1360			
85~	1813	23.9	796	43.9	1017			
95~	1234	16.2	563	45.6	671			
**Gender**								
Male	3509	46.2	1291	36.8	2218		48.964	<0.001
Female	4086	53.8	1827	44.7	2259			
**Residence**								
Town	4433	58.4	1759	39.7	2674		8.302	0.004
Countryside	3162	41.6	1359	43.0	1803			
**Marital status**								
Without spouse	3800	50.0	1755	46.2	2045		82.733	<0.001
With spouse	3795	50.0	1363	35.9	2432			
**Activities of daily living**								
Impaired	1140	15.0	569	49.9	571		43.500	<0.001
Normal	6455	85.0	2549	39.5	3906			
**Self-rated health status**								
Good	3716	48.9	926	24.9	2790	75.1	915.768	<0.001
Fair	2932	38.6	1505	51.3	1427	48.7		
Poor	947	12.5	687	72.5	260	27.5		
**Hearing status**								
Difficult	5230	68.9	1128	47.7	1237	52.3	62.615	<0.001
Normal	2365	31.1	1990	38.0	3240	62.0		
**Serious illness in the past two years**								
Yes	5505	72.5	946	45.3	1144	54.7	21.117	<0.001
No	2090	27.5	2172	39.5	3333	60.5		
**Education level**								
Illiterates	3197	42.1	1542	48.2	1655	51.8	121.099	<0.001
Primary and junior high school	3558	46.8	1299	36.5	2259	63.5		
High school or above	840	11.1	277	33.0	563	67.0		
**Occupation**								
general practitioners	5325	70.1	2369	44.5	2956	55.5	104.569	<0.001
intermediate practitioners	1227	16.2	454	37.0	773	63.0		
senior practitioners	1043	13.7	295	28.3	748	71.7		
**Economic status**								
Poor	733	9.7	486	66.3	247	33.7	367.291	<0.001
Fair	5331	70.2	2255	42.3	3076	57.7		
Good	1531	20.2	377	24.6	1154	75.4		

Among the participants, 42.1% had illiterate education, 46.8% had primary or junior high school education, and 11.1% had senior high school education or above. 70.1% of the participants were ordinary practitioners, 16.2% were intermediate practitioners, and 13.7% were senior practitioners. 9.7%, 70.2% and 20.2% of the participants rated their economic status as poor, fair and good, respectively. SES composite scores in this study ranged from −1.46 to 2.59. Cognitive function scores ranged from 0 to 30, with an average score of 26.2 ± 5.1. The score of lifestyles was between 0 and 6, with an average score of 3. 9 ± 1.2. The score of social participation ranged from 11 to 55, with an average score of 23.1 ± 7.2.

The mean CES-D-10 score of all the participants was 9.6 ± 5.4. Using ≥10 as the cut-off score, depressive symptoms were identified in 41.1% of the participants. The chi-square test results revealed that age, gender, residence, marital status, activities of daily living, self-rated health status, hearing status, serious illness in the past two years, education level, occupation, and economic status were each associated with differences in the prevalence of depressive symptoms (*P* < 0.001).

### Correlation analysis

Pearson correlation was used to analyze the correlations between CES-D score, socioeconomic status, cognitive function score, lifestyle score, and social participation score (Table 2). The results showed that CES-D score was negatively correlated with socioeconomic status (*r* = −0.216, *P* < 0.001), cognitive function score (*r* = −0.202, *P* < 0.001), lifestyle score (*r* = −0.203, *P* < 0.001), and social participation score (*r* = −0.198, *P* < 0.001). Socioeconomic status was positively correlated with cognitive function score (*r* = 0.289, *P* < 0.001), lifestyle score (*r* = 0.276, *P* < 0.001) and social participation score (*r* = 0.335, *P* < 0.001). In addition, cognitive function score, lifestyle score and social participation score were positively correlated, and the correlation coefficient was between 0.160 and 0.436 (*P* < 0.001).

**Table 2:** Correlation analysis (*n* = 7595).

Variables	Depression	SES	Cognitive function	Lifestyle	Social participation
**Depression**	1				
**SES**	−0.216^***^	1			
**Cognitive function**	−0.202^***^	0.289^***^	1		
**Lifestyle**	−0.203^***^	0.276^***^	0.160^***^	1	
**Social participation**	−0.198^***^	0.335^***^	0.436^***^	0.244^***^	1

### The mediating role of cognitive function, lifestyle and social participation in the effect of SES on depressive symptoms

The results of the mediation model analysis (Table 3) showed that SES negatively affected depression in older adults (β = −0.887, *P* < 0.001) after controlling for sociodemographic variables and health status variables (Model Ⅰ), and SES still negatively affected depression in older adults (β = −0.541, *P* < 0.001) after adding cognitive function, lifestyle and social participation as mediating variables (Model Ⅴ). Moreover, SES positively affected cognitive function (β = 0.835, *P* < 0.001), lifestyle (β = 0.345, *P* < 0.001), and social participation (β = 1.359, *P* < 0.001) among older adults (Models Ⅱ-Ⅳ). Cognitive function (β = −0.085, *P* < 0.001), lifestyle (β = −0.512, *P* < 0.001) and social participation (β = −0.050, *P* < 0.001) negatively affected depression in older adults (Model Ⅴ). Cognitive function positively affected lifestyle (β = 0.020, *P* < 0.001) and social participation (β = 0.225, *P* < 0.001) in older adults (Models Ⅲ-Ⅳ).

**Table 3:** Mediation model of the effect of socioeconomic status on cognitive function, lifestyle, social participation, and depressive symptoms in older adults. (*n* = 7595).

Variables	Model Ⅰ	Model Ⅱ	Model Ⅲ	Model Ⅳ	Model Ⅴ
**SES**	−0.887^***^	0.835^***^	0.345^***^	1.359^***^	−0.541^***^
**Cognitive function**	–	–	0.020^***^	0.225^***^	−0.085^***^
**Lifestyle**	–	–	–	0.716^***^	−0.512^***^
**Social participation**	–	–	–	–	−0.050^***^
**Age**	−0.001	−1.440^***^	−0.028	−1.763^***^	−0.259^***^
**Gender**	0.166	−0.904^***^	0.580^***^	0.337^*^	0.404^**^
**Residence**	−0.375^**^	−0.152	−0.119^***^	−0.232	−0.468^***^
**Marital status**	−0.679^***^	0.265^*^	−0.030	0.754^***^	−0.597^***^
**ADL**	−0.374^*^	2.646^***^	0.034	3.329^***^	0.094
**Self-rated health status**	3.139^***^	−0.555^***^	−0.166^***^	−0.438^***^	2.967^***^
**Hearing status**	0.541^***^	−1.809^***^	−0.007	−0.139	0.336^*^
**Serious illness in the past two years**	0.049	0.149	0.045	−0.292	0.075
***R*** ^**2**^	0.221	0.346	0.148	0.363	0.243
***F***	239.589^***^	445.542^***^	132.118^***^	392.288^***^	202.996^***^

Note: ^*^P < 0.05, ^**^P < 0.01, ^***^P < 0.001. Values in the table are standardized regression coefficients. Models Ⅰ and Ⅴ used depression as the dependent variable. Model Ⅱ included cognitive function as the dependent variable. Model Ⅲ included lifestyle as the dependent variable. Model Ⅳ included social participation as the dependent variable.

**Fig 2:**
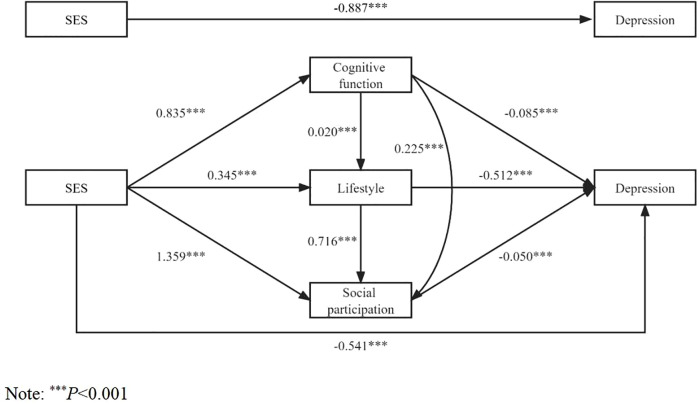
Path analysis of the influence of socioeconomic status on depression in the elderly.

Mediation test results (Table 4) showed that the total indirect effect of SES on depression in older adults through cognitive function, lifestyle and social participation was significant (relative mediating effect = 39.0%, β = −0.346, 95%CI: −0.398 ~ −0.294), and the direct effect was also significant (relative mediating effect = 61.0%, β = −0.541, 95%CI: −0.670 ~ −0.412). In the indirect effect, SES has a significant mediating effect on depression in older adults through “cognitive function” (relative mediating effect = 8.0%, β = −0.071, 95%CI: −0.095 ~ −0.048), “lifestyle” (relative mediating effect = 19.9%, β = −0.177, 95%CI: −0.213 ~ −0.140) and “social participation” (relative mediating effect = 7.6%, β = −0.068, 95%CI: −0.095 ~ −0.042), respectively. Moreover, the influence of SES on depression was also significantly mediated by “cognitive function → lifestyle” (relative mediating effect = 1.0%, β = −0.009, 95%CI: −0.012 ~ −0.006), “cognitive function → social participation” (relative mediating effect = 1.0%, β = −0.009, 95%CI: −0.014 ~ −0.006), “lifestyle → social participation” (relative mediating effect = 1.4%, β = −0.012, 95%CI: −0.018 ~ −0.007) and “cognitive function → lifestyle →social participation” (relative mediating effect = 0.1%, β = −0.001, 95%CI: −0.001 ~ −0.001) respectively. Fig 2 shows the path analysis of SES influencing depression in older adults.

**Table 4:** Testing of mediating effects of cognitive function, lifestyle, and social participation.

Type of effects	Coefficient	Boot 95%*CI*		Relative mediating effect
		Lower	Upper	
**Total effect**	−0.887	−1.009	−0.765	100.0%
**Direct effect**	−0.541	−0.670	−0.412	61.0%
**Total indirect effect**	−0.346	−0.398	−0.294	39.0%
**SES→cognitive function→depression**	−0.071	−0.095	−0.048	8.0%
**SES→lifestyle→depression**	−0.177	−0.213	−0.140	19.9%
**SES→social participation→depression**	−0.068	−0.095	−0.042	7.6%
**SES→cognitive function→lifestyle→depression**	−0.009	−0.012	−0.006	1.0%
**SES→cognitive function→social participation→depression**	−0.009	−0.014	−0.006	1.0%
**SES→lifestyle→social participation→depression**	−0.012	−0.018	−0.007	1.4%
**SES→cognitive function→lifestyle→social participation→depression**	−0.001	−0.001	−0.001	0.1%

Note: Effect values in the table are standardized effect values.

This study conducts some robust tests to confirm the accuracy of the estimation outcomes, including the stratified analysis by residence and gender. The results indicating that the mediating effects of cognitive function, lifestyle, and social participation are consistent across different living environments and populations. Tables 5– present the inspection findings.

**Table 5:** Male mediating effects of cognitive function, lifestyle, and social participation.

Type of effects	Coefficient	Boot 95%*CI*		Relative mediating effect
		Lower	Upper	
**Total effect**	−0.175	−0.216	−0.141	100.0%
**Total indirect effect**	−0.081	−0.104	−0.069	46.2%
**SES→cognitive function→depression**	−0.015	−0.025	−0.011	8.7%
**SES→lifestyle→depression**	−0.038	−0.056	−0.029	21.7%
**SES→social participation→depression**	−0.020	−0.035	−0.012	11.2%
**SES→cognitive function→lifestyle→depression**	−0.002	−0.003	−0.001	1.0%
**SES→cognitive function→social participation→depression**	−0.003	−0.005	−0.001	1.5%
**SES→lifestyle→social participation→depression**	−0.004	−0.007	−0.002	2.1%
**SES→cognitive function→lifestyle→social participation→depression**	0.000	0.000	0.000	0.1%

Note: Effect values in the table are standardized effect values.

**Table 6:** Female mediating effects of cognitive function, lifestyle, and social participation.

Type of effects	Coefficient	Boot 95%*CI*		Relative mediating effect
		Lower	Upper	
**Total effect**	−0.173	−0.277	−0.170	100.0%
**Total indirect effect**	−0.051	−0.088	−0.041	29.5%
**SES→cognitive function→depression**	−0.012	−0.026	−0.006	6.8%
**SES→lifestyle→depression**	−0.022	−0.040	−0.014	12.9%
**SES→social participation→depression**	−0.01	−0.032	−0.004	7.0%
**SES→cognitive function→lifestyle→depression**	−0.001	−0.003	−0.001	0.7%
**SES→cognitive function→social participation→depression**	−0.002	−0.004	0.000	1.0%
**SES→lifestyle→social participation→depression**	−0.002	−0.005	−0.001	1.2%
**SES→cognitive function→lifestyle→social participation→depression**	0.000	0.000	0.000	0.1%

**Table 7:** Urban mediating effects of cognitive function, lifestyle, and social participation.

Type of effects	Coefficient	Boot 95%*CI*		Relative mediating effect
		Lower	Upper	
**Total effect**	−0.179	−0.232	−0.139	100.0%
**Total indirect effect**	−0.077	−0.102	−0.058	43.2%
**SES→cognitive function→depression**	−0.014	−0.050	−0.006	7.9%
**SES→lifestyle→depression**	−0.042	−0.062	−0.026	23.2%
**SES→social participation→depression**	−0.014	−0.025	−0.008	8.0%
**SES→cognitive function→lifestyle→depression**	−0.002	−0.003	−0.001	1.1%
**SES→cognitive function→social participation→depression**	−0.002	−0.004	−0.001	1.2%
**SES→lifestyle→social participation→depression**	−0.003	−0.005	−0.001	1.7%
**SES→cognitive function→lifestyle→social participation→depression**	0.000	0.000	0.000	0.1%

**Table 8:** Rural mediating effects of cognitive function, lifestyle, and social participation.

Type of effects	Coefficient	Boot 95%*CI*		Relative mediating effect
		Lower	Upper	
**Total effect**	−0.164	−0.328	−0.177	100.0%
**Total indirect effect**	−0.043	−0.085	−0.045	26.3%
**SES→cognitive function→depression**	−0.009	−0.022	−0.005	5.7%
**SES→lifestyle→depression**	−0.016	−0.044	−0.012	9.5%
**SES→social participation→depression**	−0.013	−0.038	−0.006	8.2%
**SES→cognitive function→lifestyle→depression**	−0.001	−0.003	−0.001	0.6%
**SES→cognitive function→social participation→depression**	−0.002	−0.005	−0.001	1.2%
**SES→lifestyle→social participation→depression**	−0.002	−0.005	−0.001	1.2%
**SES→cognitive function→lifestyle→social participation→depression**	0.000	0.000	0.000	0.1%

## Discussion

The present study demonstrates that 41.1% of older adults reported depressive symptoms, with lower socioeconomic status (SES) significantly increasing the likelihood of depression. Cognitive function, lifestyle, and social participation were identified as partial mediators, with lifestyle exerting the most pronounced effect. These findings underscore both the direct influence of SES and the indirect mechanisms through which cognitive, behavioral, and social factors contribute to depressive symptoms in later life.

These findings suggest that the mental health status of older adults in China remains a critical concern [32]. The high prevalence of depressive symptoms observed in this study may be partly attributable to rapid socioeconomic transformations, including the weakening of traditional family structures and the increasing number of “empty-nest” elderly, both of which may exacerbate psychological vulnerability in later life [33].

Our findings confirm an inverse association between SES and depressive symptoms in older adults, consistent with prior research. Ng et al. reported higher odds of depressive symptoms among low-SES older adults in Singapore [34]. Lei et al. identified a strong SES gradient in depressive symptoms among Chinese older adults [35]. This finding is also consistent with the association between other health outcomes (such as physical health and cognitive function) and SES [36], highlighting those socioeconomic inequalities contribute to health disparities, particularly in mental health. According to emotion cognition theory, subjective deprivation is the cognitive link between social environment and mental disorders, stemming from perceived relative income disparities [37]. Subjective deprivation due to perceived relative income disparity among low-income groups appears to be a key contributor to depressive symptoms. Low-SES older adults may experience greater stress and negative emotions, increasing their vulnerability to mental health issues. In addition, limited access to adequate healthcare services and lower quality of routine care may exacerbate illness burden and depressive symptoms [38]. Therefore, appropriate policies and interventions should be implemented to reduce the SES related inequalities in depressive symptoms among older adults.

This study further demonstrates that SES influences depressive symptoms through cognitive function, lifestyle, and social participation. Nutakor et al. found that higher education and income are associated with better cognitive function [39]. There are two possible explanations for the association between SES and cognitive function: the Brain Reserve Capacity Theory (higher education enhances brain reserve) [40]and the behavioral regulatory role of education (promoting healthy lifestyles that support cognitive function) [41].The complex interplay between cognitive function and depression is well-documented, and the prevalence of major depression in patients with Alzheimer’s disease is about 17% [42], and it’s even higher in patients with subcortical dementia [7]. The clinical manifestations of depression intersect with dementia in older adults, and they are difficult to distinguish from each other. The results of this study show that cognitive function can negatively predict depression in older adults, that is, older adults with better cognitive function are less likely to be depressed, which also partially confirms the positive association between cognitive function and depression in older adults.

Studies in developed countries such as the United States have shown that groups with higher SES have healthier lifestyles [43]. Lower SES groups may face disadvantages in risk perception, learning efficacy, and social capital, leading to less healthy behaviors. Unhealthy lifestyles such as low diet quality, smoking, excessive alcohol consumption, lack of exercise and insomnia are associated with depressive symptoms in older adults [44–46]. The underlying physiological mechanism may be that unhealthy lifestyle (poor eating habits, insufficient sleep, and exposure to chemicals and pollutants) may potentially disturb the hypothalamic-pituitary-adrenal axis, increase cortisol, and cause low-grade systemic inflammation and oxidative stress [47]. Both neuroendocrine disorders and inflammation have been implicated in the etiology of depression [48].

Previous studies have also supported the positive association between SES and social participation [18]. Berkman et al.’s path mode [49]suggests that social structural factors (e.g., SES) shape social network size, which in turn facilitates social participation, support, and resource access. Thus, the higher SES group has a wider social network and their social participation is higher. The Activity Theory [50]emphasizes that regular social participation is critical for maintaining self-esteem, psychological satisfaction, and physical health in older adults, highlighting the mental health benefits of active social engagement.

While our findings support the pathway from SES to depressive symptoms via cognitive and behavioral mediators, interpretation warrants caution due to the possibility of bidirectional relationships. The ‘Health Selection Hypothesis’ posits that early-onset depressive symptoms or cognitive decline might lead to downward socioeconomic mobility or social withdrawal, thereby inflating the observed associations. Although we controlled for baseline health status, the cross-sectional design limits our ability to completely rule out reverse causality—for instance, depression itself serves as a barrier to maintaining a healthy lifestyle and social participation. Furthermore, potential survival bias must be considered. As the CLHLS targets the oldest-old, individuals with low SES and severe depression might have had higher mortality rates and were thus excluded from the sample. This selection bias implies that our study might actually underestimate the true adverse impact of low SES on depression, as the ‘frailest’ participants are missing. Additionally, unlike Western contexts, the protective effect of SES in China might be moderated by traditional family support systems, which act as a buffer. Future longitudinal studies using cross-lagged panel models are needed to disentangle these complex temporal dynamics.

Several limitations of this study should be addressed. First, the cross-sectional data used in this study make it difficult to test the inference of causality, we must acknowledge in the discussion that the association between SES and depression is likely bidirectional. And CLHLS survey focused on individuals aged 65 and older. Individuals who died prematurely due to low SES and related health problems (including severe depression) were not included in the study sample. Secondly, the data in this study are all from questionnaires, which may cause investigation bias. Thirdly, this study did not include other covariates, such as drugs, and more in-depth research should be carried out in the next step. Finally, we included lifestyle and social participation as a whole in the mediation analysis, and did not conduct a detailed analysis of specific relevant variables. Therefore, longitudinal studies after 2018 should be conducted to further explore the causal association between SES and depressive symptoms in older adults and other related mechanisms, including cognitive decline, unhealthy lifestyle, and adverse childhood experiences.

## Conclusion

In conclusion, this study found that SES directly and indirectly influences depressive symptoms in older adults through cognitive function, lifestyle, and social participation. These findings underscore the importance of addressing socioeconomic disparities, promoting healthy behaviors, and encouraging social engagement to improve mental health in aging populations. The results provide an evidence base for designing targeted interventions and policy measures aimed at supporting older adults with low SES.
